# The relationship between severe maternal morbidity and a risk of postpartum readmission among Korean women: a nationwide population-based cohort study

**DOI:** 10.1186/s12884-020-2820-7

**Published:** 2020-03-06

**Authors:** Jin Young Nam, Eun-Cheol Park

**Affiliations:** 1 Research Institute of Asian Women, Sookmyung Women’s University, 47 Na-gil 36 Cheongpa-ro, Yongsan-gu, Seoul, 04309 Republic of Korea; 2 Department of Preventive Medicine, Yonsei University College of Medicine, Seoul, Republic of Korea; 3 Institute of Health Services Research, Yonsei University, 50 Yonsei-ro, Seodaemun-gu, Seoul, 03722 Republic of Korea

**Keywords:** Postpartum readmission, Severe maternal morbidity, Mode of delivery, Cohort study

## Abstract

**Background:**

As the rate of cesarean section delivery has increased, the incidence of severe maternal morbidity continues to increase. Severe maternal morbidity is associated with high medical costs, extended length of hospital stay, and long-term rehabilitation. However, there is no evidence whether severe maternal morbidity affects postpartum readmission. Therefore, this study aimed to determine the relationship between severe maternal morbidity and postpartum readmission.

**Methods:**

This nationwide population-based cohort study used the Korean National Health Insurance Service-National Sample cohort of 90,035 delivery cases between January 2003 and November 2013. The outcome variable was postpartum readmission until 6 weeks after the first date of delivery in the hospital. Another variable of interest was the occurrence of severe maternal morbidity, which was determined using the Center for Disease Control and Prevention’s algorithm. The Cox proportional hazard model was used to assess the association between postpartum readmission and severe maternal morbidity after all covariates were adjusted.

**Results:**

The overall incidence of postpartum readmission was 2041 cases (0.95%) of delivery. Women with severe maternal morbidity had an approximately 2.4 times higher risk of postpartum readmission than those without severe maternal morbidity (hazard ratio 2.36, 95% confidence interval 1.75–3.19). In addition, compared with reference group, women who were aged 20–30 years, nulliparous, and delivered in a tertiary hospital were at high risk of postpartum readmission.

**Conclusions:**

Severe maternal morbidity was related to the risk of postpartum readmission. Policy makers should provide a quality indicator of postpartum maternal health care and improve the quality of intrapartum care.

## Introduction

Compared with the maternal mortality ratio, severe maternal morbidity (SMM) is a useful indicator for the evaluation and improvement of maternal health services [1], and it use as such an indicator has increased. SMM is defined as unintended outcomes of the process of labor and delivery that result in significant short-term or long-term consequences to a woman’s health [2]. Although SMM is rare, it is associated with high medical costs, extended length of hospital stay, and long-term rehabilitation [3]. To improve maternal health care, it is necessary to determine a relevant measurement of SMM, find preventable factors for SMM, and monitor the quality of maternal care during postpartum. Unplanned hospital readmission is used as a quality metric in medical and surgical health care [4], and it focuses on quality improvement and the reduction of medical costs [5]. Although the readmission rate is used as a quality indicator, postpartum readmission rates are not used as a quality metric in obstetric care [5]. Postpartum readmission was used as one of the indicators of postpartum maternal morbidity [6], but few researchers suggested that it could be a quality indicator in obstetric care [5].

Previous studies identified the risk factors for SMM including maternal age [7], family income [8], residing in disadvantaged areas [9], employment status [10], comorbidities [11], multiple births [12], cesarean section (CS) delivery [11, 13, 14], and hospital volume [15]. Although several studies identified which factors affected postpartum readmission [5, 16–18], most of them focused on the relationship between postpartum readmission and the mode of delivery, and CS was especially associate with postpartum readmission [16–18]. CS has been commonly considered as a safe and low-risk procedure [18] with a high incidence rate. In the United States (US), the rate of CS slightly declined in 2016 (31.9%), but in the recent 7 years, it accounted for more than 32% of all deliveries [19]. In South Korea, CS was the fourth most frequent type of total surgery in 2017 [20]; approximately 45% of women had CS, and it use has been increasing [21]. However, CS was significantly related to the risk of SMM [11, 13, 14], double-fold higher medical costs [22], and a high risk of postpartum readmission [16]. The evidence of the association between SMM and postpartum readmission is almost non-existent. This study aimed to determine whether SMM is related to the risk of postpartum readmission.

## Methods

### Data source, study design, and population

This retrospective cohort study used the Korean National Health Insurance Service-National Sample Cohort (NHIS-NSC) from 2002 to 2013. The NHIS-NSC included approximately 1 million Koreans’ information since 2002. This database collected 2.5% (*n* = 1,025,340) of total Koreans using a stratified random sampling method in 2002. It included Koreans’ age, sex, residential area, type of health insurance, household income decile, medical diagnosis and procedure, prescription drugs, and individual total medical costs. The NHIS-NSC can track patient and clinical characteristics over time, reveal epidemiological causes of disease, and be used to develop health policies. These data did not include direct personal identifiers but included the unique de-identified numbers of patients’ sociodemographic and medical information. Furthermore, individuals with unique de-identified numbers were connected to mortality information from the Korean National Statistical Office [23].

This study included all women aged 15 years or older and less than 50 years who had a delivery in the hospital and were enrolled for at least a year before childbirth to 42 days after childbirth between January 1, 2003 and November 19, 2013. Childbirth was defined as any inpatient admission records with a delivery-related diagnosis or procedural code for vaginal delivery or CS among women who delivered full-term babies (i.e., ≥37 weeks’ gestational age). We excluded women who had preterm births, those whose delivery hospitalization was more than 42 days, or those who died during delivery hospitalization. The total population was 90,035 cases of delivery between January 2003 and November 2013.

### Postpartum readmission

The outcome variable was postpartum readmission within 42 days after the first date of delivery hospitalization. We excluded women who were continuously admitted 42 days after delivery hospitalization and those who died during delivery hospitalization.

### Severe maternal morbidity

SMM was determined using an algorithm of the Centers for Disease Control and Prevention (CDC), which consisted of 25 indicators for SMM including 18 diagnoses and 7 procedure codes [3, 24]. The indicators for SMM were either serious complications of pregnancy or delivery. Eighteen indicators were identified using diagnostic codes converted from the International Classification of Diseases, Ninth Revision (ICD-9) Clinical Modification ICD-9-CM to the International Classification of Diseases, Tenth Revision (ICD-10), and 7 indicators were procedure codes. We defined women who met at least 1 of the 25 indicators for SMM during delivery hospitalization has having SMM.

### Covariates

Maternal characteristics included maternal age (15–49 years), household income level (quintile), type of insurance (self-employed insured, employee insured, or medical aid), residential area (rural or urban), working status (working or not working), mode of delivery (spontaneous vaginal [SV] delivery, instrumental delivery, or CS), parity (1, 2, or 3+), number of births (singleton or twin), and comorbidities during pregnancy (0 or + 1). Hospital characteristics included the type of hospital (primary, secondary, or tertiary hospital), profit status (public or private), teaching status (non-teaching or teaching), and year of delivery year, which was used as an adjustable variable.

### Statistical analysis

We estimated the distribution of study participants who delivered between 2003 and 2013. The association between SMM and postpartum readmission was analyzed using time-to-event methods. Kaplan Meier curves were generated to compare unadjusted postpartum readmission rates according to groups of SMM. Cox proportional hazard models were used to calculate the adjusted hazard ratios (HRs) and 95% confidence intervals (CIs) for the relationship between postpartum readmission and SMM. The proportionality assumption was tested using Schoenfeld-like residuals. In the subgroup analysis, the Cox proportional hazard model was used to determine whether SMM affected postpartum readmission by the mode of delivery. All statistical analyses were conducted using SAS 9.4 (SAS Institute, Inc., Cary, NC, USA). The level of significance was set at *P* < 0.05.

## Results

Table 1 shows the general characteristics of the study participants according to SMM (see supplementary Table S1). Of the 90,035 women included in this study, 2041 (2.27%) had SMM during the delivery hospitalization. The overall postpartum readmission was 0.95%. Among women with SMM, postpartum readmission was recorded in 2.45, and 0.92% of those without SMM had postpartum readmission.

**Table 1:** General characteristic of study population

	Total (*N* = 90,035)		No SMM (*N* = 87,994)		SMM (*N* = 2041)	
	N	(%)	N	(%)	N	(%)
Postpartum readmission						
No	89,178	(99.05)	87,187	(99.08)	1991	(97.55)
Yes	857	(0.95)	807	(0.92)	50	(2.45)
*Maternal characteristics*						
Maternal age (years)						
15–19	298	(0.33)	284	(0.32)	14	(0.69)
20–24	4151	(4.61)	4055	(4.61)	96	(4.70)
25–29	28,212	(31.33)	27,737	(31.52)	475	(23.27)
30–34	42,410	(47.10)	41,492	(47.15)	918	(44.98)
35–39	13,170	(14.63)	12,739	(14.48)	431	(21.12)
40+	1794	(1.99)	1687	(1.92)	107	(5.24)
Income level						
1Q	8472	(9.41)	8244	(9.37)	228	(11.17)
2Q	13,185	(14.64)	12,881	(14.64)	304	(14.89)
3Q	23,587	(26.20)	23,074	(26.22)	513	(25.13)
4Q	29,342	(32.59)	28,708	(32.62)	634	(31.06)
5Q	15,449	(17.16)	15,087	(17.15)	362	(17.74)
Type of insurance						
Self-employed insured	26,225	(29.13)	25,558	(29.05)	667	(32.68)
Employee insured	63,527	(70.56)	62,167	(70.65)	1360	(66.63)
Medical aid	283	(0.31)	269	(0.31)	14	(0.69)
Residential area						
Rural	26,636	(29.58)	25,976	(29.52)	660	(32.34)
Urban	63,399	(70.42)	62,018	(70.48)	1381	(67.66)
Working status						
Work	25,055	(27.83)	24,500	(27.84)	555	(27.19)
Not work	64,980	(72.17)	63,494	(72.16)	1486	(72.81)
Mode of delivery						
Spontaneous vaginal delivery	31,990	(35.53)	31,617	(35.93)	373	(18.28)
Instrumental delivery	24,648	(27.38)	24,229	(27.53)	419	(20.53)
Cesarean section delivery	33,397	(37.09)	32,148	(36.53)	1249	(61.20)
Parity						
1 (Nulliparous)	60,081	(66.73)	58,547	(66.54)	1534	(75.16)
2	26,573	(29.51)	26,126	(29.69)	447	(21.90)
3+	3381	(3.76)	3321	(3.77)	60	(2.94)
Twin birth status						
Singleton	88,944	(98.79)	87,013	(98.89)	1931	(94.61)
Twin	1091	(1.21)	981	(1.11)	110	(5.39)
Comorbidities during pregnancy						
0	73,743	(81.90)	72,500	(82.39)	1243	(60.90)
1+	16,292	(18.10)	15,494	(17.61)	798	(37.10)
*Hospital characteristics*						
Type of hospital						
Primary	42,542	(47.25)	41,973	(47.70)	569	(27.88)
Secondary	38,200	(42.43)	37,578	(42.71)	622	(30.48)
Tertiary	9293	(10.32)	8443	(9.59)	850	(41.65)

*SMM* severe maternal morbidity

**Fig. 1:**
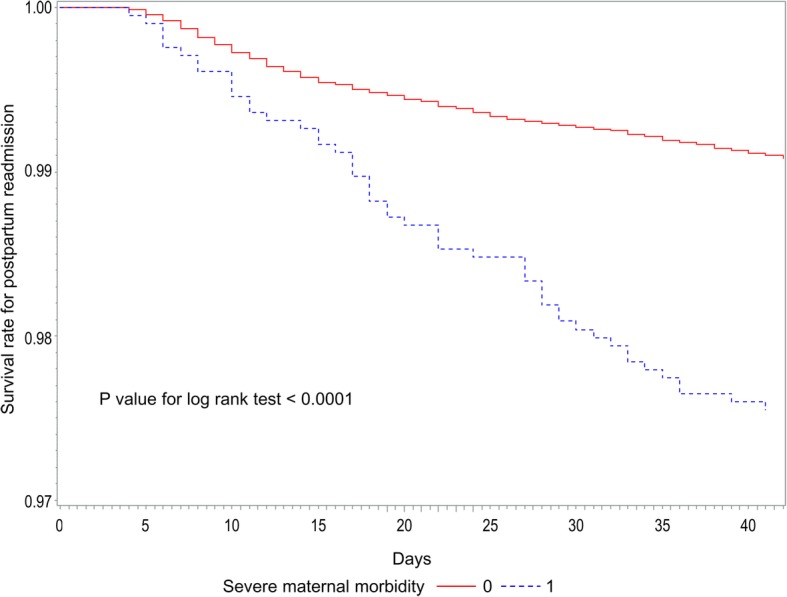
Kaplan Meier curves for the incidence of postpartum readmission according to severe maternal morbidity

Figure 1 presents the Kaplan Meier curve for the comparison of the unadjusted postpartum readmission rates according to SMM. The incidence of postpartum readmission was significantly higher in women with SMM than in those without SMM (*P* < 0.0001).

Table 2 presents the results of the Cox proportional hazard analysis (see supplementary Table S2 for full model). The risk of postpartum readmission was higher in women with SMM than in those without SMM (HR: 2.36, 95% CI: 1.75–3.19). In addition, compared with the reference group, women who in their 20s (20–24 years: HR 1.38, 95% CI 1.02–1.88; 25–29 years: HR: 1.29, 95% CI: 1.10–1.51), those who were nulliparous (HR: 1.59, 95% CI: 1.04–2.45), and those who delivered in a tertiary hospital (HR: 1.55, 95% CI: 1.19–2.02) were at high risk of postpartum readmission.

**Table 2:** The association between the incidence of postpartum readmission and risk factors

			Postpartum readmission	
	Total	N	Hazard Ratio	95% CI
Severe maternal morbidity				
No	87,994	807	1.00	
Yes	2041	50	2.35	1.75–3.17
Maternal age (years)				
15–19	298	6	2.13	0.92–4.88
20–24	4151	50	1.38	1.02–1.88
25–29	28,212	308	1.29	1.10–1.51
30–34	42,410	355	1.00	
35–39	13,170	116	1.02	0.83–1.27
40+	1794	22	1.26	0.82–1.96
Income level				
1Q	8472	87	1.01	0.77–1.34
2Q	13,185	132	1.00	0.78–1.27
3Q	23,587	216	0.75	0.76–1.18
4Q	29,342	282	1.03	0.84–1.27
5Q	15,449	140	1.00	
Type of insurance				
Self-employed insured	26,225	256	1.05	0.91–1.22
Employee insured	63,527	598	1.00	
Medical aid	283	3	0.90	0.28–2.88
Residential area				
Rural	26,636	281	1.13	0.98–1.31
Urban	63,399	576	1.00	
Mode of delivery				
Spontaneous vaginal delivery	31,990	282	1.00	
Instrumental delivery	24,648	255	1.08	0.91–1.28
Cesarean section delivery	33,397	320	1.01	0.86 1.20
Parity				
1 (Nulliparous)	60,081	629	1.59	1.03–2.44
2	26,573	206	1.23	0.79–1.90
3+	3381	22	1.00	
Comorbidities during pregnancy				
0	73,743	676	1.00	
1+	16,292	181	1.07	0.91–1.27
Type of hospital				
Primary	42,542	400	1.09	0.94–1.27
Secondary	38,200	327	1.00	
Tertiary	9293	130	1.55	1.19–2.02

Adjusted all covariates

**Fig. 2:**
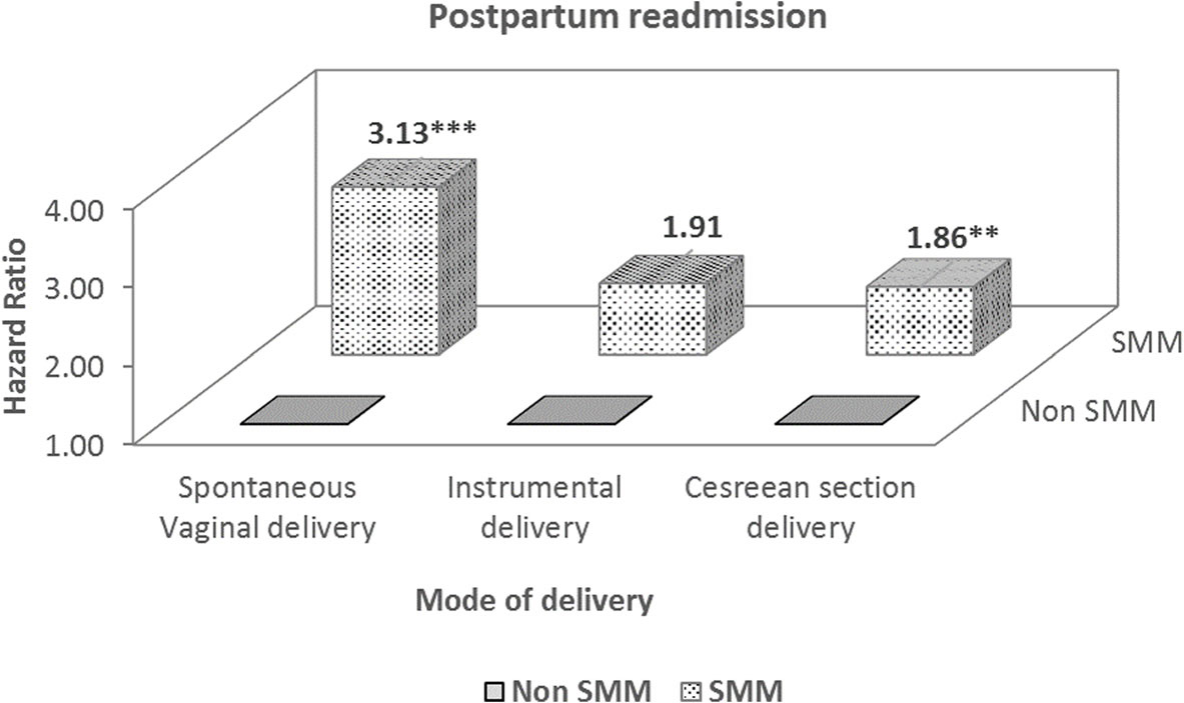
Subgroup analysis for the association between severe maternal morbidity and postpartum readmission by mode of delivery. * < 0.05, ** < 0.01, *** < 0.001. SMM: severe maternal morbidity

Figure 2 shows the results of the subgroup analysis between postpartum readmission and SMM by the mode of delivery. Women with SMM and spontaneous vaginal (SV) delivery or CS were more likely to have a high risk of postpartum readmission than those without SMM (SV delivery, HR: 3.31, 95% CI: 1.71–5.79; CS, HR: 1.86, 95% CI: 1.26–2.77). There was marginally no statistical significant association between women with SMM and instrumental delivery.

## Discussion

The current study found that SMM increases the risk of postpartum readmission. Additionally, women in their 20s, nulliparous women, and women who delivered in a tertiary hospital were associated with the risk of postpartum readmission. Although there was no statistically significant association between the type of delivery and postpartum readmission, women with SMM and SV delivery or CS were at high risk of postpartum readmission, which was a statistically significant association. To our knowledge, this population-based cohort study is the first one to determine the relationship between SMM and postpartum readmission using nationwide cohort data.

Herein, the overall incidence of SMM was 2.27%, which was similar and different compared to that reported previously. Previous studies reported an SMM incidence of 2–2.5% [9, 25, 26], whereas others reported that of 0.3–1.7% [11, 14, 27]. This difference could be because participants had different maternal health conditions in their communities or countries, and different indicators for SMM were used. Several US studies have shown that the incidence of SMM is 2–2.5% because the study populations included individuals of the same nationality, although their races were different, and they used the same SMM indicators, such as CDC’s algorithm [9, 25, 26]. In contrast, other studies that have shown a low incidence of SMM used clinical indicators of SMM such as blood cell transfusion units [11, 27], ICU admission [11, 27], or opinion of the treating obstetrician [14, 27]. Moreover, this may explain the difference in the participants’ nationality, which affects maternal health conditions based on various cultures, communities, and countries’ healthcare systems. However, CDC’s algorithm for SMM was used in this study; the incidence of SMM was similar to that reported in previous studies that used CDC’s algorithm, even though most of our study population was Asian. Therefore, the used indicators might affect the incidence rate of SMM. Moreover, the overall incidence of postpartum readmission was 0.95%, which was less than that in previous studies. One Canadian study reported that the incidence of postpartum readmission was 1.8% within 60 days after initial discharge [18], whereas other studies in the US noted that 1.2% or 1.72–2.16% of women experienced postpartum readmission within 6 weeks after delivery from 2004 to 2011 [5, 16, 17]. This difference might be because of the different health care systems or obstetric care, or the different evaluation of quality of care could affect the incidence of postpartum readmission. Further, our methods of calculating postpartum readmission might have underestimated the incidence.

This study confirmed that SMM and the risk of postpartum readmission were significantly related. Women with SMM had a 2.35 times higher risk of postpartum readmission than those without SMM. Additionally, mothers aged 20–24 and 25–29 years had a higher risk of postpartum readmission than those aged 30–34 years, and nulliparous women had a higher risk of postpartum readmission than did multiparous women. There was no evidence of the cause of this relationship, but we can hypothesize the etiology. Previous studies have revealed that the relationship between age and the risk of SMM is J-shaped, which means that young and older women have a high risk of SMM [28, 29]. These studies also showed that nulliparous women have an increased risk of SMM. In the current study, younger women had several risks of SMM, especially mothers in their teens or early twenties who are more likely to be nulliparous, have a low income level [28], and have less adequate prenatal care access [29]; that is, young women might have significant risks of SMM, and therefore, younger age might be associated with the incidence of postpartum readmission. Although women with SMM recover their symptoms of SMM, it is necessary to continuously monitor them during postpartum. However, the health care system tended to avoid long-term hospitalization. When patients do not have severe complications, early hospital discharge is recommended, and new patients are hospitalized because of the health care system’s profit structure. Another possible reason for the lack of evidence is the reduction of human resources. Staff deficiency, a lack of resources and preparedness for emergent situations, and less experienced physicians during off-hour periods are expected to be associated with poorer quality and safety [30, 31]. Deficient human resources might cause poor quality of maternal care and lead to adverse health outcomes [30], as well as increasing readmission during postpartum.

For the mode of delivery, we could not find a statistical significant association with postpartum readmission. Liu and colleagues showed that total CS had a 2.18 times higher risk of postpartum readmission than vaginal delivery [18]. Clapp et al. reported that CS had a 1.43 times higher risk of all-cause postpartum readmission than vaginal delivery [5], but this finding resulted from unadjusted covariates. In another study, CS had a statistically significant association with postpartum readmission when adjusted by year, patient demographics, and hospital characteristics, but when maternal comorbidities were adjusted, the relationship between CS and postpartum readmission became insignificant [16]. Herein, CS did not have a statistically significant association with postpartum readmission compared with SV delivery because we adjusted SMM and all other control variables. Women with SMM had a high risk of CS [11, 13, 14]; if we controlled for SMM, the risky health condition of maternity would be uniform and only women with a relatively healthy pregnancy might have remained. Furthermore, we excluded women with an extremely long-term delivery admission and those who died during delivery hospitalization. CS might have had an insignificant association with postpartum readmission because we did not include high-risk women. However, in the subgroup analysis, women who with SMM had a 3.13 times and 1.86 times higher risk of postpartum readmission by SV delivery and CS, respectively. Regardless of the mode of delivery, SMM was a high-risk factor for postpartum readmission; especially, if women with SMM had SV delivery, the risk was significantly increased compared with other modes of delivery.

Several limitations of this study must be discussed. First, the incidence of postpartum readmission can change depending on the method of calculating readmission based on the administrative data. Although our readmission rate was underestimated, it might more accurately reflect the real-world readmission rate. Second, we did not estimate cause-specific postpartum readmission but included all-cause postpartum readmission. The aspects of cause-specific readmission might be overestimated. It might have been better to determine which factors were more likely to affect the risk of postpartum readmission. Further research is needed to determine the specific causes of postpartum readmission. Third, we used administrative data that did not include relevant clinical data on the severity of disease; therefore, we did not identify the severity of SMM. Moreover, there was a problem with conversion of the ICD-9 procedural codes. The ICD-10 codes did not include the procedural codes; therefore, we converted the ICD-9 procedural codes to match the EDI codes. During this process, some codes did not match the EDI codes; hence, some procedure-based SMM indicators might have been less precise.

Nevertheless, our study has several strengths. First, this is a unique study of SMM and postpartum readmission. Until recently, there has been little evidence regarding SMM and postpartum readmission with any maternal, clinical, and provisional indicators. Our research might provide important evidence for use in future maternal health care. Second, this study had a population-based design and included long-term follow-up and nationwide data; therefore, the results could be representative of the nation. Lastly, this database was continuously updated for 12 years without periods of interruption.

## Conclusions

This study founded that severe maternal morbidity was related to the risk of postpartum readmission. Policy makers should consider introducing policies to improve the quality of maternity care during the intrapartum and postpartum periods to minimize the incidence of SMM and reduce postpartum readmission. Since we did not determine the causes for postpartum readmission, more research on this subject and whether sub-indicators of SMM affect specific causes of postpartum readmission is needed. This information would provide further evidence that could support individual-level and population-level policies for improving maternal health outcomes and quality of care for childbirth.

## Supplementary information

**Additional file 1: Table S1.** General characteristic of study population. **Table S2.** The association between the incidence of postpartum readmission and risk factors.

## Data Availability

The Korean National Health Insurance Service-National Sample Cohort data that support the findings of this study are available from the National Health Insurance Data Sharing Service (https://nhiss.nhis.or.kr/bd/ab/bdaba022eng.do) but restrictions apply to the availability of these data, which were used under license for the current study, and so are not publicly available. Data are however available from the authors upon reasonable request and with permission of the National Health Insurance Data Sharing Service.

## Abbreviations

CDC: Centers for Disease Control and Prevention
CIs: Confidence intervals
CS: Cesarean section
HRs: Hazard ratios
ICD-10: International Classification of Diseases, 10th revision
ICD-9: International Classification of Diseases, 9th Revision
NHIS-NSC: Korean National Health Insurance Service-National Sample Cohort
SMM: Severe maternal morbidity
SV: Spontaneous vaginal
US: United States
