# Supplementary material for: The relationship between severe maternal morbidity and a risk of postpartum readmission among Korean women: a nationwide population-based cohort study
Source: BMC Pregnancy Childbirth. 2020 Mar 6;20:148. doi: 10.1186/s12884-020-2820-7 (PMC7060630; doi:10.1186/s12884-020-2820-7)
Supplement: Supplementary file 1 — Additional file 1: Table S1. General characteristic of study population. Table S2. The association between the incidence of postpartum readmission and risk factors. [file 12884_2020_2820_MOESM1_ESM.docx]

Table S1. General characteristic of study population.

|  |  | **Total** (N = 90,035) | | **No SMM** (N = 87,994) | | **SMM** (N = 2,041) | |
| --- | --- | --- | --- | --- | --- | --- | --- |
|  |  | **N** | **(%)** | **N** | **(%)** | **N** | **(%)** |
| Postpartum readmission | |  |  |  |  |  |  |
|  | No | 89178 | (99.05) | 87187 | (99.08) | 1991 | (97.55) |
|  | Yes | 857 | (0.95) | 807 | (0.92) | 50 | (2.45) |
| ***Maternal characteristics*** | |  |  |  |  |  |  |
| Maternal age (years) | |  |  |  |  |  |  |
|  | 15-19 | 298 | (0.33) | 284 | (0.32) | 14 | (0.69) |
|  | 20-24 | 4151 | (4.61) | 4055 | (4.61) | 96 | (4.70) |
|  | 25-29 | 28212 | (31.33) | 27737 | (31.52) | 475 | (23.27) |
|  | 30-34 | 42410 | (47.10) | 41492 | (47.15) | 918 | (44.98) |
|  | 35-39 | 13170 | (14.63) | 12739 | (14.48) | 431 | (21.12) |
|  | 40+ | 1794 | (1.99) | 1687 | (1.92) | 107 | (5.24) |
| Income level | |  |  |  |  |  |  |
|  | 1Q | 8472 | (9.41) | 8244 | (9.37) | 228 | (11.17) |
|  | 2Q | 13185 | (14.64) | 12881 | (14.64) | 304 | (14.89) |
|  | 3Q | 23587 | (26.20) | 23074 | (26.22) | 513 | (25.13) |
|  | 4Q | 29342 | (32.59) | 28708 | (32.62) | 634 | (31.06) |
|  | 5Q | 15449 | (17.16) | 15087 | (17.15) | 362 | (17.74) |
| Type of insurance | |  |  |  |  |  |  |
|  | Self-employed insured | 26225 | (29.13) | 25558 | (29.05) | 667 | (32.68) |
|  | Employee insured | 63527 | (70.56) | 62167 | (70.65) | 1360 | (66.63) |
|  | Medical aid | 283 | (0.31) | 269 | (0.31) | 14 | (0.69) |
| Residential area | |  |  |  |  |  |  |
|  | Rural | 26636 | (29.58) | 25976 | (29.52) | 660 | (32.34) |
|  | Urban | 63399 | (70.42) | 62018 | (70.48) | 1381 | (67.66) |
| Working status | |  |  |  |  |  |  |
|  | Work | 25055 | (27.83) | 24500 | (27.84) | 555 | (27.19) |
|  | Not work | 64980 | (72.17) | 63494 | (72.16) | 1486 | (72.81) |
| Mode of delivery | |  |  |  |  |  |  |
|  | Spontaneous vaginal delivery | 31990 | (35.53) | 31617 | (35.93) | 373 | (18.28) |
|  | Instrumental delivery | 24648 | (27.38) | 24229 | (27.53) | 419 | (20.53) |
|  | Cesarean section delivery | 33397 | (37.09) | 32148 | (36.53) | 1249 | (61.20) |
| Parity | |  |  |  |  |  |  |
|  | 1 (Nulliparous) | 60081 | (66.73) | 58547 | (66.54) | 1534 | (75.16) |
|  | 2 | 26573 | (29.51) | 26126 | (29.69) | 447 | (21.90) |
|  | 3+ | 3381 | (3.76) | 3321 | (3.77) | 60 | (2.94) |
| Twin birth status | |  |  |  |  |  |  |
|  | Singleton | 88944 | (98.79) | 87013 | (98.89) | 1931 | (94.61) |
|  | Twin | 1091 | (1.21) | 981 | (1.11) | 110 | (5.39) |
| Comorbidities during pregnancy | |  |  |  |  |  |  |
|  | 0 | 73743 | (81.90) | 72500 | (82.39) | 1243 | (60.90) |
|  | 1+ | 16292 | (18.10) | 15494 | (17.61) | 798 | (37.10) |
| ***Hospital characteristics*** | |  |  |  |  |  |  |
| Type of hospital | |  |  |  |  |  |  |
|  | Primary | 42542 | (47.25) | 41973 | (47.70) | 569 | (27.88) |
|  | Secondary | 38200 | (42.43) | 37578 | (42.71) | 622 | (30.48) |
|  | Tertiary | 9293 | (10.32) | 8443 | (9.59) | 850 | (41.65) |
| Profit status | |  |  |  |  |  |  |
|  | Public | 394 | (0.44) | 382 | (0.43) | 12 | (0.59) |
|  | Private | 89641 | (99.56) | 87612 | (99.57) | 2029 | (99.41) |
| Teaching status | |  |  |  |  |  |  |
|  | No | 85743 | (95.23) | 84194 | (95.68) | 1549 | (75.89) |
|  | Yes | 4292 | (4.77) | 3800 | (4.32) | 492 | (24.11) |
| Year | |  |  |  |  |  |  |
|  | 2003 | 8382 | (9.31) | 8181 | (9.30) | 201 | (9.85) |
|  | 2004 | 8356 | (9.28) | 8138 | (9.25) | 218 | (10.68) |
|  | 2005 | 8074 | (8.97) | 7865 | (8.94) | 209 | (10.24) |
|  | 2006 | 8110 | (9.01) | 7931 | (9.01) | 179 | (8.77) |
|  | 2007 | 8895 | (9.88) | 8678 | (9.86) | 217 | (10.63) |
|  | 2008 | 8160 | (9.06) | 8005 | (9.10) | 155 | (7.59) |
|  | 2009 | 7425 | (8.25) | 7252 | (8.24) | 173 | (8.48) |
|  | 2010 | 7792 | (8.65) | 7606 | (8.64) | 186 | (9.11) |
|  | 2011 | 8474 | (9.41) | 8300 | (9.43) | 174 | (8.53) |
|  | 2012 | 8622 | (9.58) | 8437 | (9.59) | 185 | (9.06) |
|  | 2013 | 7745 | (8.60) | 7601 | (8.64) | 144 | (7.06) |

Table S2. The association between the incidence of postpartum readmission and risk factors.

|  |  |  |  | **Postpartum readmission** | | | |
| --- | --- | --- | --- | --- | --- | --- | --- |
|  |  | **Total** | **N** | **Hazard Ratio** | **95% CI** | | |
| Severe maternal morbidity | |  |  |  |  |  |  |
|  | No | 87994 | 807 | 1.00 |  |  |  |
|  | Yes | 2041 | 50 | 2.35 | 1.75 | - | 3.17 |
| ***Maternal characteristics*** | |  |  |  |  |  |  |
| Maternal age (years) | |  |  |  |  |  |  |
|  | 15-19 | 298 | 6 | 2.13 | 0.92 | - | 4.88 |
|  | 20-24 | 4151 | 50 | 1.38 | 1.02 | - | 1.88 |
|  | 25-29 | 28212 | 308 | 1.29 | 1.10 | - | 1.51 |
|  | 30-34 | 42410 | 355 | 1.00 |  |  |  |
|  | 35-39 | 13170 | 116 | 1.02 | 0.83 | - | 1.27 |
|  | 40+ | 1794 | 22 | 1.26 | 0.82 | - | 1.96 |
| Income level | |  |  |  |  |  |  |
|  | 1Q | 8472 | 87 | 1.01 | 0.77 | - | 1.34 |
|  | 2Q | 13185 | 132 | 1.00 | 0.78 | - | 1.27 |
|  | 3Q | 23587 | 216 | 0.75 | 0.76 | - | 1.18 |
|  | 4Q | 29342 | 282 | 1.03 | 0.84 | - | 1.27 |
|  | 5Q | 15449 | 140 | 1.00 |  |  |  |
| Type of insurance | |  |  |  |  |  |  |
|  | Self-employed insured | 26225 | 256 | 1.05 | 0.91 | - | 1.22 |
|  | Employee insured | 63527 | 598 | 1.00 |  |  |  |
|  | Medical aid | 283 | 3 | 0.90 | 0.28 | - | 2.88 |
| Residential area | |  |  |  |  |  |  |
|  | Rural | 26636 | 281 | 1.13 | 0.98 | - | 1.31 |
|  | Urban | 63399 | 576 | 1.00 |  |  |  |
| Working status | |  |  |  |  |  |  |
|  | Work | 25055 | 259 | 1.11 | 0.95 | - | 1.30 |
|  | Not work | 64980 | 598 | 1.00 |  |  |  |
| Mode of delivery | |  |  |  |  |  |  |
|  | Spontaneous vaginal delivery | 31990 | 282 | 1.00 |  |  |  |
|  | Instrumental delivery | 24648 | 255 | 1.08 | 0.91 | - | 1.28 |
|  | Cesarean section delivery | 33397 | 320 | 1.01 | 0.86 |  | 1.20 |
| Parity | |  |  |  |  |  |  |
|  | 1 (Nulliparous) | 60081 | 629 | 1.59 | 1.03 | - | 2.44 |
|  | 2 | 26573 | 206 | 1.23 | 0.79 | - | 1.90 |
|  | 3+ | 3381 | 22 | 1.00 |  |  |  |
| Twin birth status | |  |  |  |  |  |  |
|  | Singleton | 88935 | 845 | 1.00 |  |  |  |
|  | Twin | 1100 | 12 | 0.86 | 0.47 | - | 1.58 |
| Comorbidities during pregnancy | |  |  |  |  |  |  |
|  | 0 | 73743 | 676 | 1.00 |  |  |  |
|  | 1+ | 16292 | 181 | 1.07 | 0.91 | - | 1.27 |
| ***Hospital characteristics*** | |  |  |  |  |  |  |
| Type of hospital | |  |  |  |  |  |  |
|  | Primary | 42542 | 400 | 1.09 | 0.94 | - | 1.27 |
|  | Secondary | 38200 | 327 | 1.00 |  |  |  |
|  | Tertiary | 9293 | 130 | 1.55 | 1.19 | - | 2.02 |
| Profit status | |  |  |  |  |  |  |
|  | Public | 394 | 5 | 1.17 | 0.48 | - | 2.86 |
|  | Private | 89641 | 852 | 1.00 |  |  |  |
| Teaching status | |  |  |  |  |  |  |
|  | No | 85743 | 800 | 1.09 | 0.76 | - | 1.55 |
|  | Yes | 4292 | 57 | 1.00 |  |  |  |
| Year | |  |  |  |  |  |  |
|  | 2003 | 8382 | 76 | 1.00 |  |  |  |
|  | 2004 | 8356 | 75 | 1.03 | 0.74 | - | 1.41 |
|  | 2005 | 8074 | 81 | 1.18 | 0.86 | - | 1.62 |
|  | 2006 | 8110 | 67 | 1.01 | 0.72 | - | 1.40 |
|  | 2007 | 8895 | 92 | 1.28 | 0.94 | - | 1.75 |
|  | 2008 | 8160 | 85 | 1.32 | 0.96 | - | 1.81 |
|  | 2009 | 7425 | 67 | 1.16 | 0.82 | - | 1.62 |
|  | 2010 | 7792 | 73 | 1.22 | 0.88 | - | 1.70 |
|  | 2011 | 8474 | 82 | 1.27 | 0.92 | - | 1.75 |
|  | 2012 | 8622 | 97 | 1.48 | 1.08 | - | 2.02 |
|  | 2013 | 7745 | 62 | 1.07 | 0.75 | - | 1.51 |
